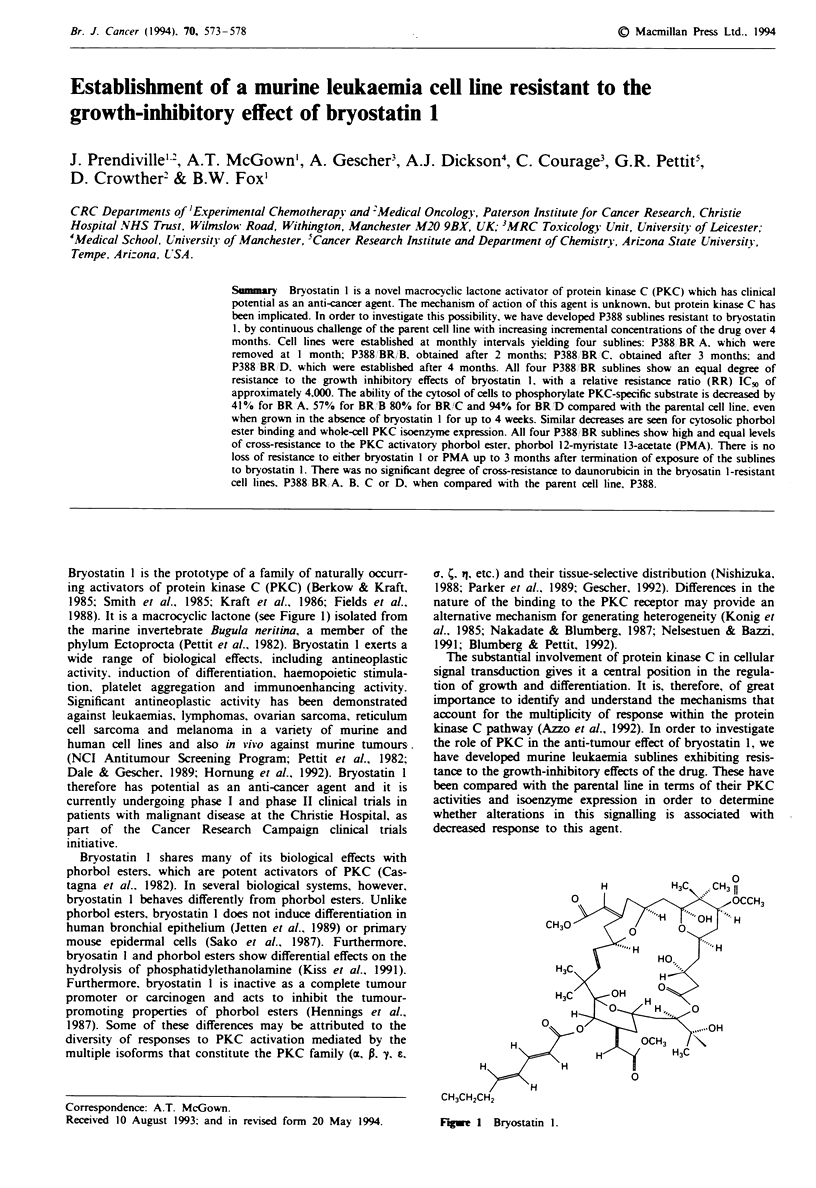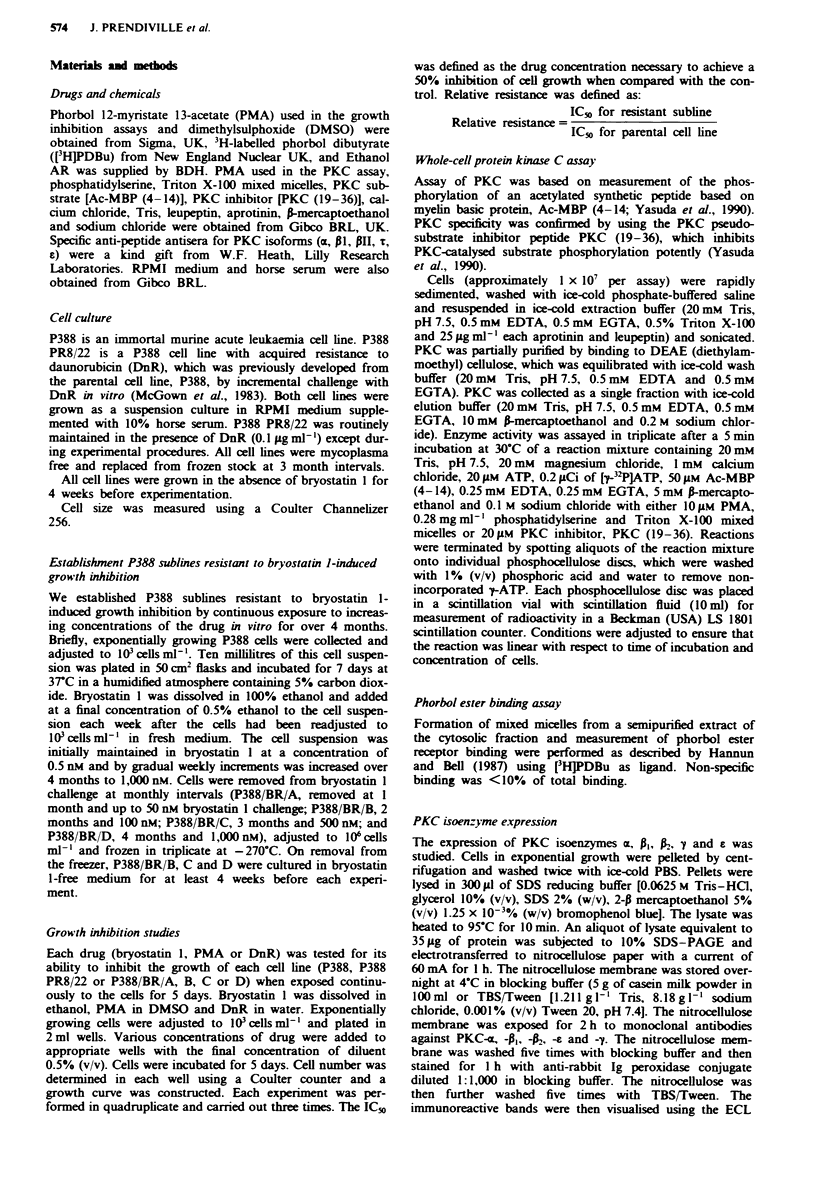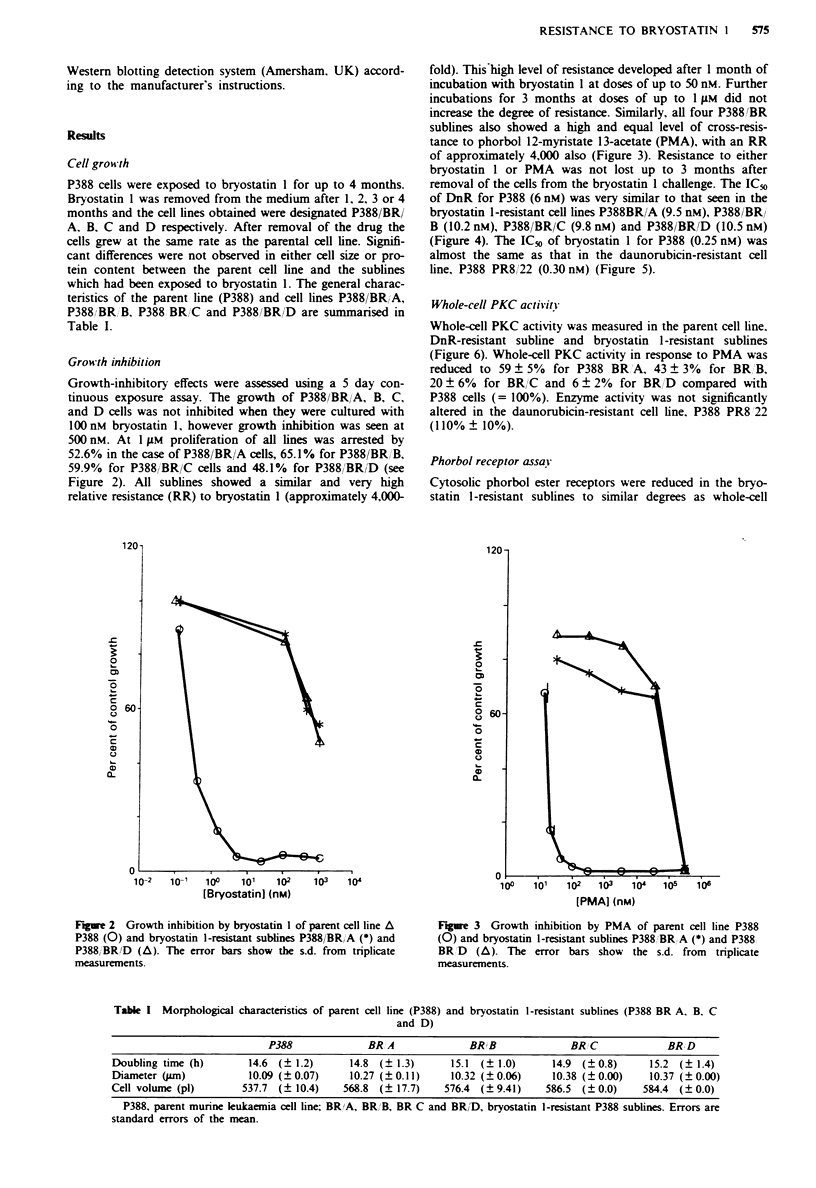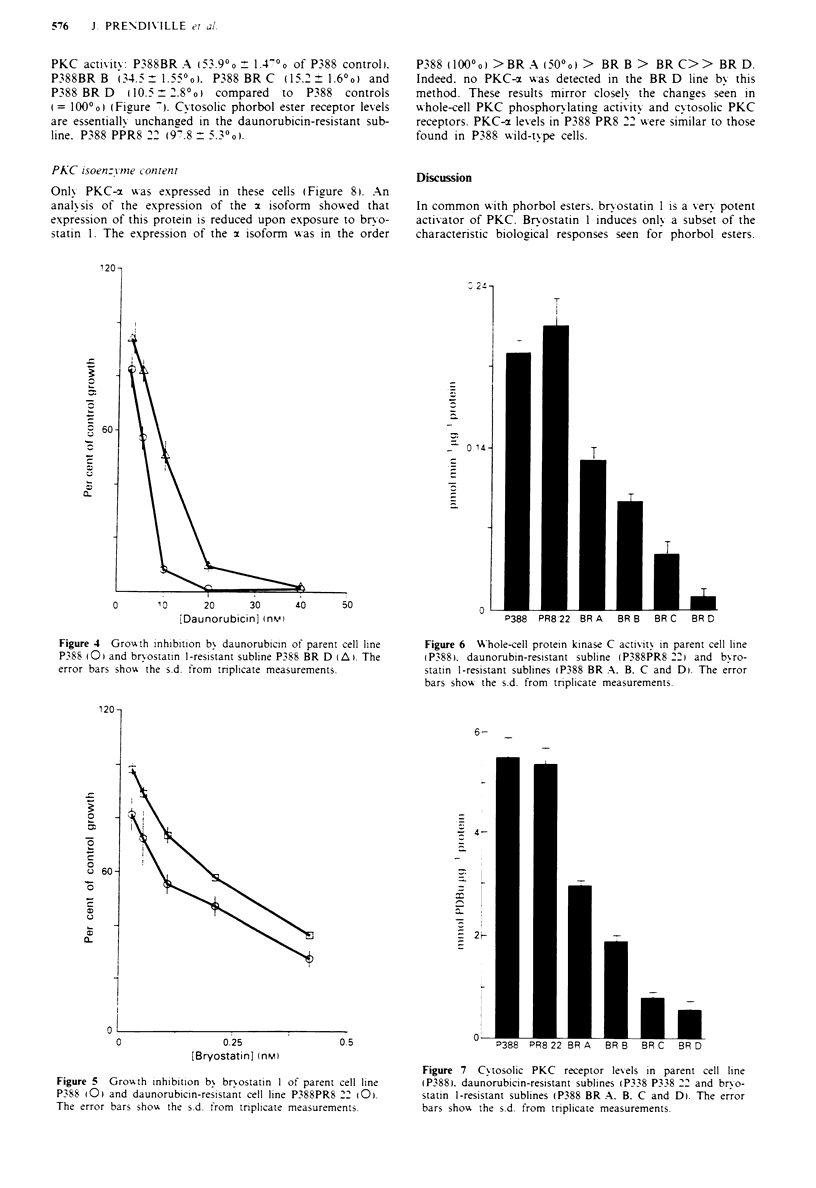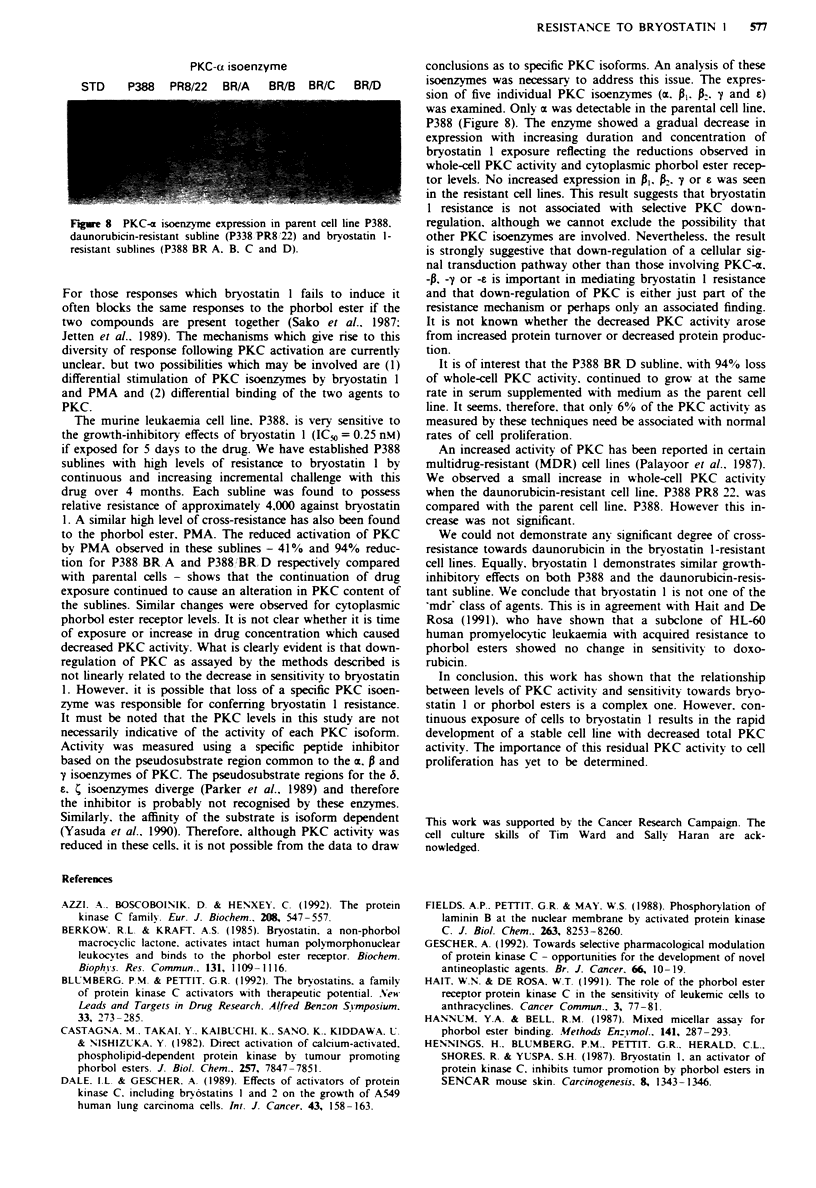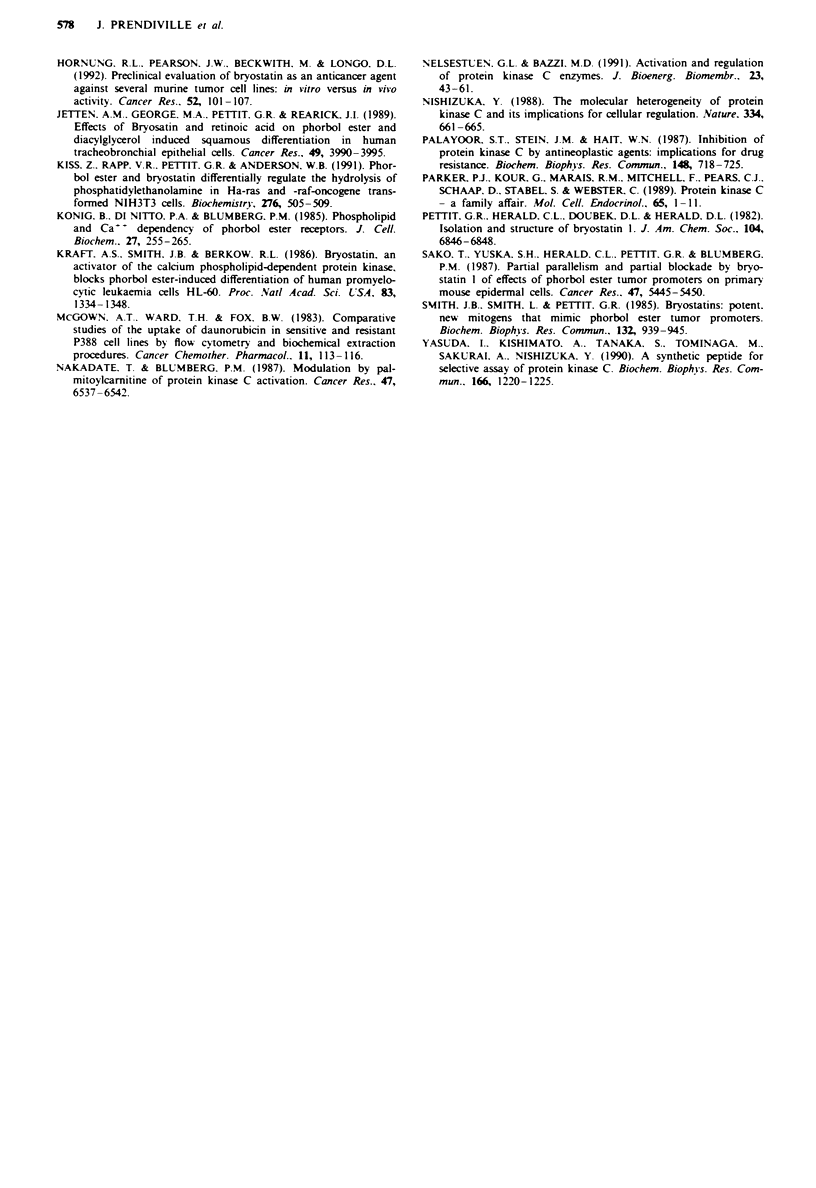# Establishment of a murine leukaemia cell line resistant to the growth-inhibitory effect of bryostatin 1.

**DOI:** 10.1038/bjc.1994.353

**Published:** 1994-10

**Authors:** J. Prendiville, A. T. McGown, A. Gescher, A. J. Dickson, C. Courage, G. R. Pettit, D. Crowther, B. W. Fox

**Affiliations:** CRC Department of Experimental Chemotherapy, Paterson Institute for Cancer Research, Christie Hospital NHS Trust, Withington, UK.

## Abstract

**Images:**


Br. J. Cancer (1994), **70**, 573-578 © Macmillan Press Ltd., 1994

### Establishment of a murine leukaemia cell line resistant to the growth-inhibitory effect of bryostatin 1

J. Prendiville[1,2], A.T. McGown[1], A. Gescher[3], A.J. Dickson[4], C. Courage[3], G.R. Pettit[5], D. Crowther[2] & B.W. Fox[1]

*CRC Departments of [1]Experimental Chemotherapy and [2]Medical Oncology, Paterson Institute for Cancer Research, Christie Hospital NHS Trust, Wilmslow Road, Withington, Manchester M20 9BX, UK; [3]MRC Toxicology Unit, University of Leicester; [4]Medical School, University of Manchester, [5]Cancer Research Institute and Department of Chemistry, Arizona State University, Tempe, Arizona, USA.*

**Summary** Bryostatin 1 is a novel macrocyclic lactone activator of protein kinase C (PKC) which has clinical potential as an anti-cancer agent. The mechanism of action of this agent is unknown, but protein kinase C has been implicated. In order to investigate this possibility, we have developed P388 sublines resistant to bryostatin 1, by continuous challenge of the parent cell line with increasing incremental concentrations of the drug over 4 months. Cell lines were established at monthly intervals yielding four sublines: P388/BR/A, which were removed at 1 month; P388/BR/B, obtained after 2 months; P388/BR/C, obtained after 3 months; and P388/BR/D, which were established after 4 months. All four P388/BR sublines show an equal degree of resistance to the growth inhibitory effects of bryostatin 1, with a relative resistance ratio (RR) $IC_{50}$ of approximately 4,000. The ability of the cytosol of cells to phosphorylate PKC-specific substrate is decreased by 41% for BR/A, 57% for BR/B 80% for BR/C and 94% for BR/D compared with the parental cell line, even when grown in the absence of bryostatin 1 for up to 4 weeks. Similar decreases are seen for cytosolic phorbol ester binding and whole-cell PKC isoenzyme expression. All four P388/BR sublines show high and equal levels of cross-resistance to the PKC activatory phorbol ester, phorbol 12-myristate 13-acetate (PMA). There is no loss of resistance to either bryostatin 1 or PMA up to 3 months after termination of exposure of the sublines to bryostatin 1. There was no significant degree of cross-resistance to daunorubicin in the bryosatin 1-resistant cell lines, P388/BR/A, B, C or D, when compared with the parent cell line, P388.

Bryostatin 1 is the prototype of a family of naturally occurring activators of protein kinase C (PKC) (Berkow & Kraft, 1985; Smith *et al.*, 1985; Kraft *et al.*, 1986; Fields *et al.*, 1988). It is a macrocyclic lactone (see Figure 1) isolated from the marine invertebrate *Bugula neritina*, a member of the phylum Ectoprocta (Pettit *et al.*, 1982). Bryostatin 1 exerts a wide range of biological effects, including antineoplastic activity, induction of differentiation, haemopoietic stimulation, platelet aggregation and immunoenhancing activity. Significant antineoplastic activity has been demonstrated against leukaemias, lymphomas, ovarian sarcoma, reticulum cell sarcoma and melanoma in a variety of murine and human cell lines and also *in vivo* against murine tumours (NCI Antitumour Screening Program; Pettit *et al.*, 1982; Dale & Gescher, 1989; Hornung *et al.*, 1992). Bryostatin 1 therefore has potential as an anti-cancer agent and it is currently undergoing phase I and phase II clinical trials in patients with malignant disease at the Christie Hospital, as part of the Cancer Research Campaign clinical trials initiative.

Bryostatin 1 shares many of its biological effects with phorbol esters, which are potent activators of PKC (Castagna *et al.*, 1982). In several biological systems, however, bryostatin 1 behaves differently from phorbol esters. Unlike phorbol esters, bryostatin 1 does not induce differentiation in human bronchial epithelium (Jetten *et al.*, 1989) or primary mouse epidermal cells (Sako *et al.*, 1987). Furthermore, bryosatin 1 and phorbol esters show differential effects on the hydrolysis of phosphatidylethanolamine (Kiss *et al.*, 1991). Furthermore, bryostatin 1 is inactive as a complete tumour promoter or carcinogen and acts to inhibit the tumour-promoting properties of phorbol esters (Hennings *et al.*, 1987). Some of these differences may be attributed to the diversity of responses to PKC activation mediated by the multiple isoforms that constitute the PKC family (α, β, γ, ε, σ, ζ, η, etc.) and their tissue-selective distribution (Nishizuka, 1988; Parker *et al.*, 1989; Gescher, 1992). Differences in the nature of the binding to the PKC receptor may provide an alternative mechanism for generating heterogeneity (Konig *et al.*, 1985; Nakadate & Blumberg, 1987; Nelsestuen & Bazzi, 1991; Blumberg & Pettit, 1992).

The substantial involvement of protein kinase C in cellular signal transduction gives it a central position in the regulation of growth and differentiation. It is, therefore, of great importance to identify and understand the mechanisms that account for the multiplicity of response within the protein kinase C pathway (Azzo *et al.*, 1992). In order to investigate the role of PKC in the anti-tumour effect of bryostatin 1, we have developed murine leukaemia sublines exhibiting resistance to the growth-inhibitory effects of the drug. These have been compared with the parental line in terms of their PKC activities and isoenzyme expression in order to determine whether alterations in this signalling is associated with decreased response to this agent.

**Figure 1** Bryostatin 1.

Correspondence: A.T. McGown.
Received 10 August 1993; and in revised form 20 May 1994.

### Materials and methods

### *Drugs and chemicals*

Phorbol 12-myristate 13-acetate (PMA) used in the growth inhibition assays and dimethylsulphoxide (DMSO) were obtained from Sigma, UK, $^3$H-labelled phorbol dibutyrate ([$^3$H]PDBu) from New England Nuclear UK, and Ethanol AR was supplied by BDH. PMA used in the PKC assay, phosphatidylserine, Triton X-100 mixed micelles, PKC substrate [Ac-MBP (4–14)], PKC inhibitor [PKC (19–36)], calcium chloride, Tris, leupeptin, aprotinin, β-mercaptoethanol and sodium chloride were obtained from Gibco BRL, UK. Specific anti-peptide antisera for PKC isoforms (α, β1, βII, τ, ε) were a kind gift from W.F. Heath, Lilly Research Laboratories. RPMI medium and horse serum were also obtained from Gibco BRL.

### *Cell culture*

P388 is an immortal murine acute leukaemia cell line. P388 PR8/22 is a P388 cell line with acquired resistance to daunorubicin (DnR), which was previously developed from the parental cell line, P388, by incremental challenge with DnR *in vitro* (McGown *et al.*, 1983). Both cell lines were grown as a suspension culture in RPMI medium supplemented with 10% horse serum. P388 PR8/22 was routinely maintained in the presence of DnR (0.1 $\mu g\ ml^{-1}$) except during experimental procedures. All cell lines were mycoplasma free and replaced from frozen stock at 3 month intervals.

All cell lines were grown in the absence of bryostatin 1 for 4 weeks before experimentation.

Cell size was measured using a Coulter Channelizer 256.

### *Establishment P388 sublines resistant to bryostatin 1-induced growth inhibition*

We established P388 sublines resistant to bryostatin 1-induced growth inhibition by continuous exposure to increasing concentrations of the drug *in vitro* for over 4 months. Briefly, exponentially growing P388 cells were collected and adjusted to $10^3$ cells $ml^{-1}$. Ten millilitres of this cell suspension was plated in 50 $cm^2$ flasks and incubated for 7 days at 37°C in a humidified atmosphere containing 5% carbon dioxide. Bryostatin 1 was dissolved in 100% ethanol and added at a final concentration of 0.5% ethanol to the cell suspension each week after the cells had been readjusted to $10^3$ cells $ml^{-1}$ in fresh medium. The cell suspension was initially maintained in bryostatin 1 at a concentration of 0.5 nM and by gradual weekly increments was increased over 4 months to 1,000 nM. Cells were removed from bryostatin 1 challenge at monthly intervals (P388/BR/A, removed at 1 month and up to 50 nM bryostatin 1 challenge; P388/BR/B, 2 months and 100 nM; P388/BR/C, 3 months and 500 nM; and P388/BR/D, 4 months and 1,000 nM), adjusted to $10^6$ cells $ml^{-1}$ and frozen in triplicate at −270°C. On removal from the freezer, P388/BR/B, C and D were cultured in bryostatin 1-free medium for at least 4 weeks before each experiment.

### *Growth inhibition studies*

Each drug (bryostatin 1, PMA or DnR) was tested for its ability to inhibit the growth of each cell line (P388, P388 PR8/22 or P388/BR/A, B, C or D) when exposed continuously to the cells for 5 days. Bryostatin 1 was dissolved in ethanol, PMA in DMSO and DnR in water. Exponentially growing cells were adjusted to $10^3$ cells $ml^{-1}$ and plated in 2 ml wells. Various concentrations of drug were added to appropriate wells with the final concentration of diluent 0.5% (v/v). Cells were incubated for 5 days. Cell number was determined in each well using a Coulter counter and a growth curve was constructed. Each experiment was performed in quadruplicate and carried out three times. The $IC_{50}$ was defined as the drug concentration necessary to achieve a 50% inhibition of cell growth when compared with the control. Relative resistance was defined as:

$$\text{Relative resistance} = \frac{IC_{50}\text{ for resistant subline}}{IC_{50}\text{ for parental cell line}}$$

### *Whole-cell protein kinase C assay*

Assay of PKC was based on measurement of the phosphorylation of an acetylated synthetic peptide based on myelin basic protein, Ac-MBP (4–14; Yasuda *et al.*, 1990). PKC specificity was confirmed by using the PKC pseudosubstrate inhibitor peptide PKC (19–36), which inhibits PKC-catalysed substrate phosphorylation potently (Yasuda *et al.*, 1990).

Cells (approximately $1 \times 10^7$ per assay) were rapidly sedimented, washed with ice-cold phosphate-buffered saline and resuspended in ice-cold extraction buffer (20 mM Tris, pH 7.5, 0.5 mM EDTA, 0.5 mM EGTA, 0.5% Triton X-100 and 25 $\mu g\ ml^{-1}$ each aprotinin and leupeptin) and sonicated. PKC was partially purified by binding to DEAE (diethylammoethyl) cellulose, which was equilibrated with ice-cold wash buffer (20 mM Tris, pH 7.5, 0.5 mM EDTA and 0.5 mM EGTA). PKC was collected as a single fraction with ice-cold elution buffer (20 mM Tris, pH 7.5, 0.5 mM EDTA, 0.5 mM EGTA, 10 mM β-mercaptoethanol and 0.2 M sodium chloride). Enzyme activity was assayed in triplicate after a 5 min incubation at 30°C of a reaction mixture containing 20 mM Tris, pH 7.5, 20 mM magnesium chloride, 1 mM calcium chloride, 20 μM ATP, 0.2 μCi of [γ-$^{32}$P]ATP, 50 μM Ac-MBP (4–14), 0.25 mM EDTA, 0.25 mM EGTA, 5 mM β-mercaptoethanol and 0.1 M sodium chloride with either 10 μM PMA, 0.28 $mg\ ml^{-1}$ phosphatidylserine and Triton X-100 mixed micelles or 20 μM PKC inhibitor, PKC (19–36). Reactions were terminated by spotting aliquots of the reaction mixture onto individual phosphocellulose discs, which were washed with 1% (v/v) phosphoric acid and water to remove non-incorporated γ-ATP. Each phosphocellulose disc was placed in a scintillation vial with scintillation fluid (10 ml) for measurement of radioactivity in a Beckman (USA) LS 1801 scintillation counter. Conditions were adjusted to ensure that the reaction was linear with respect to time of incubation and concentration of cells.

### *Phorbol ester binding assay*

Formation of mixed micelles from a semipurified extract of the cytosolic fraction and measurement of phorbol ester receptor binding were performed as described by Hannun and Bell (1987) using [$^3$H]PDBu as ligand. Non-specific binding was $<$10% of total binding.

### *PKC isoenzyme expression*

The expression of PKC isoenzymes α, $\beta_1$, $\beta_2$, γ and ε was studied. Cells in exponential growth were pelleted by centrifugation and washed twice with ice-cold PBS. Pellets were lysed in 300 μl of SDS reducing buffer [0.0625 M Tris-HCl, glycerol 10% (v/v), SDS 2% (w/v), 2-β mercaptoethanol 5% (v/v) $1.25 \times 10^{-3}$% (w/v) bromophenol blue]. The lysate was heated to 95°C for 10 min. An aliquot of lysate equivalent to 35 μg of protein was subjected to 10% SDS-PAGE and electrotransferred to nitrocellulose paper with a current of 60 mA for 1 h. The nitrocellulose membrane was stored overnight at 4°C in blocking buffer (5 g of casein milk powder in 100 ml or TBS/Tween [1.211 $g\ l^{-1}$ Tris, 8.18 $g\ l^{-1}$ sodium chloride, 0.001% (v/v) Tween 20, pH 7.4]. The nitrocellulose membrane was exposed for 2 h to monoclonal antibodies against PKC-α, -$\beta_1$, -$\beta_2$, -ε and -γ. The nitrocellulose membrane was washed five times with blocking buffer and then stained for 1 h with anti-rabbit Ig peroxidase conjugate diluted 1:1,000 in blocking buffer. The nitrocellulose was then further washed five times with TBS/Tween. The immunoreactive bands were then visualised using the ECL

Western blotting detection system (Amersham, UK) according to the manufacturer's instructions.

### Results

### *Cell growth*

P388 cells were exposed to bryostatin 1 for up to 4 months. Bryostatin 1 was removed from the medium after 1, 2, 3 or 4 months and the cell lines obtained were designated P388/BR/A, B, C and D respectively. After removal of the drug the cells grew at the same rate as the parental cell line. Significant differences were not observed in either cell size or protein content between the parent cell line and the sublines which had been exposed to bryostatin 1. The general characteristics of the parent line (P388) and cell lines P388/BR/A, P388/BR/B, P388/BR/C and P388/BR/D are summarised in Table I.

### *Growth inhibition*

Growth-inhibitory effects were assessed using a 5 day continuous exposure assay. The growth of P388/BR/A, B, C, and D cells was not inhibited when they were cultured with 100 nM bryostatin 1, however growth inhibition was seen at 500 nM. At 1 μM proliferation of all lines was arrested by 52.6% in the case of P388/BR/A cells, 65.1% for P388/BR/B, 59.9% for P388/BR/C cells and 48.1% for P388/BR/D (see Figure 2). All sublines showed a similar and very high relative resistance (RR) to bryostatin 1 (approximately 4,000-fold). This high level of resistance developed after 1 month of incubation with bryostatin 1 at doses of up to 50 nM. Further incubations for 3 months at doses of up to 1 μM did not increase the degree of resistance. Similarly, all four P388/BR sublines also showed a high and equal level of cross-resistance to phorbol 12-myristate 13-acetate (PMA), with an RR of approximately 4,000 also (Figure 3). Resistance to either bryostatin 1 or PMA was not lost up to 3 months after removal of the cells from the bryostatin 1 challenge. The $IC_{50}$ of DnR for P388 (6 nM) was very similar to that seen in the bryostatin 1-resistant cell lines P388BR/A (9.5 nM), P388/BR/B (10.2 nM), P388/BR/C (9.8 nM) and P388/BR/D (10.5 nM) (Figure 4). The $IC_{50}$ of bryostatin 1 for P388 (0.25 nM) was almost the same as that in the daunorubicin-resistant cell line, P388 PR8/22 (0.30 nM) (Figure 5).

### *Whole-cell PKC activity*

Whole-cell PKC activity was measured in the parent cell line, DnR-resistant subline and bryostatin 1-resistant sublines (Figure 6). Whole-cell PKC activity in response to PMA was reduced to 59 ± 5% for P388 BR/A, 43 ± 3% for BR/B, 20 ± 6% for BR/C and 6 ± 2% for BR/D compared with P388 cells (= 100%). Enzyme activity was not significantly altered in the daunorubicin-resistant cell line, P388 PR8/22 (110% ± 10%).

### *Phorbol receptor assay*

Cytosolic phorbol ester receptors were reduced in the bryostatin 1-resistant sublines to similar degrees as whole-cell

**Figure 2** Growth inhibition by bryostatin 1 of parent cell line Δ P388 (O) and bryostatin 1-resistant sublines P388/BR/A (*) and P388/BR/D (Δ). The error bars show the s.d. from triplicate measurements.

**Figure 3** Growth inhibition by PMA of parent cell line P388 (O) and bryostatin 1-resistant sublines P388/BR/A (*) and P388/BR/D (Δ). The error bars show the s.d. from triplicate measurements.

**Table I** Morphological characteristics of parent cell line (P388) and bryostatin 1-resistant sublines (P388 BR/A, B, C and D)

| | *P388* | *BR/A* | *BR/B* | *BR/C* | *BR/D* |
|---|---|---|---|---|---|
| Doubling time (h) | 14.6 (± 1.2) | 14.8 (± 1.3) | 15.1 (± 1.0) | 14.9 (± 0.8) | 15.2 (± 1.4) |
| Diameter (μm) | 10.09 (± 0.07) | 10.27 (± 0.11) | 10.32 (± 0.06) | 10.38 (± 0.00) | 10.37 (± 0.00) |
| Cell volume (pl) | 537.7 (± 10.4) | 568.8 (± 17.7) | 576.4 (± 9.41) | 586.5 (± 0.0) | 584.4 (± 0.0) |

P388, parent murine leukaemia cell line; BR/A, BR/B, BR/C and BR/D, bryostatin 1-resistant P388 sublines. Errors are standard errors of the mean.

PKC activity: P388BR A (53.9% ± 1.47% of P388 control), P388BR B (34.5 ± 1.55%), P388 BR C (15.2 ± 1.6%) and P388 BR D (10.5 ± 2.8%) compared to P388 controls (= 100%) (Figure 7). Cytosolic phorbol ester receptor levels are essentially unchanged in the daunorubicin-resistant subline, P388 PPR8 22 (97.8 ± 5.3%).

*PKC isoenzyme content*

Only PKC-α was expressed in these cells (Figure 8). An analysis of the expression of the α isoform showed that expression of this protein is reduced upon exposure to bryostatin 1. The expression of the α isoform was in the order P388 (100%) > BR A (50%) > BR B > BR C >> BR D. Indeed, no PKC-α was detected in the BR D line by this method. These results mirror closely the changes seen in whole-cell PKC phosphorylating activity and cytosolic PKC receptors. PKC-α levels in P388 PR8 22 were similar to those found in P388 wild-type cells.

### Discussion

In common with phorbol esters, bryostatin 1 is a very potent activator of PKC. Bryostatin 1 induces only a subset of the characteristic biological responses seen for phorbol esters.

**Figure 4** Growth inhibition by daunorubicin of parent cell line P388 (O) and bryostatin 1-resistant subline P388 BR D (Δ). The error bars show the s.d. from triplicate measurements.

**Figure 5** Growth inhibition by bryostatin 1 of parent cell line P388 (O) and daunorubicin-resistant cell line P388PR8 22 (O). The error bars show the s.d. from triplicate measurements.

**Figure 6** Whole-cell protein kinase C activity in parent cell line (P388), daunorubin-resistant subline (P388PR8 22) and byrostatin 1-resistant sublines (P388 BR A, B, C and D). The error bars show the s.d. from triplicate measurements.

**Figure 7** Cytosolic PKC receptor levels in parent cell line (P388), daunorubicin-resistant sublines (P338 P338 22 and bryostatin 1-resistant sublines (P388 BR A, B, C and D). The error bars show the s.d. from triplicate measurements.

**Figure 8** PKC-α isoenzyme expression in parent cell line P388, daunorubicin-resistant subline (P338/PR8/22) and bryostatin 1-resistant sublines (P388 BR A, B, C and D).

For those responses which bryostatin 1 fails to induce it often blocks the same responses to the phorbol ester if the two compounds are present together (Sako *et al*., 1987; Jetten *et al*., 1989). The mechanisms which give rise to this diversity of response following PKC activation are currently unclear, but two possibilities which may be involved are (1) differential stimulation of PKC isoenzymes by bryostatin 1 and PMA and (2) differential binding of the two agents to PKC.

The murine leukaemia cell line, P388, is very sensitive to the growth-inhibitory effects of bryostatin 1 ($IC_{50}$ = 0.25 nM) if exposed for 5 days to the drug. We have established P388 sublines with high levels of resistance to bryostatin 1 by continuous and increasing incremental challenge with this drug over 4 months. Each subline was found to possess relative resistance of approximately 4,000 against bryostatin 1. A similar high level of cross-resistance has also been found to the phorbol ester, PMA. The reduced activation of PKC by PMA observed in these sublines – 41% and 94% reduction for P388/BR/A and P388/BR/D respectively compared with parental cells – shows that the continuation of drug exposure continued to cause an alteration in PKC content of the sublines. Similar changes were observed for cytoplasmic phorbol ester receptor levels. It is not clear whether it is time of exposure or increase in drug concentration which caused decreased PKC activity. What is clearly evident is that down-regulation of PKC as assayed by the methods described is not linearly related to the decrease in sensitivity to bryostatin 1. However, it is possible that loss of a specific PKC isoenzyme was responsible for conferring bryostatin 1 resistance. It must be noted that the PKC levels in this study are not necessarily indicative of the activity of each PKC isoform. Activity was measured using a specific peptide inhibitor based on the pseudosubstrate region common to the α, β and γ isoenzymes of PKC. The pseudosubstrate regions for the δ, ε, ζ isoenzymes diverge (Parker *et al*., 1989) and therefore the inhibitor is probably not recognised by these enzymes. Similarly, the affinity of the substrate is isoform dependent (Yasuda *et al*., 1990). Therefore, although PKC activity was reduced in these cells, it is not possible from the data to draw conclusions as to specific PKC isoforms. An analysis of these isoenzymes was necessary to address this issue. The expression of five individual PKC isoenzymes (α, $\beta_1$, $\beta_2$, γ and ε) was examined. Only α was detectable in the parental cell line, P388 (Figure 8). The enzyme showed a gradual decrease in expression with increasing duration and concentration of bryostatin 1 exposure reflecting the reductions observed in whole-cell PKC activity and cytoplasmic phorbol ester receptor levels. No increased expression in $\beta_1$, $\beta_2$, γ or ε was seen in the resistant cell lines. This result suggests that bryostatin 1 resistance is not associated with selective PKC down-regulation, although we cannot exclude the possibility that other PKC isoenzymes are involved. Nevertheless, the result is strongly suggestive that down-regulation of a cellular signal transduction pathway other than those involving PKC-α, -β, -γ or -ε is important in mediating bryostatin 1 resistance and that down-regulation of PKC is either just part of the resistance mechanism or perhaps only an associated finding. It is not known whether the decreased PKC activity arose from increased protein turnover or decreased protein production.

It is of interest that the P388/BR/D subline, with 94% loss of whole-cell PKC activity, continued to grow at the same rate in serum supplemented with medium as the parent cell line. It seems, therefore, that only 6% of the PKC activity as measured by these techniques need be associated with normal rates of cell proliferation.

An increased activity of PKC has been reported in certain multidrug-resistant (MDR) cell lines (Palayoor *et al*., 1987). We observed a small increase in whole-cell PKC activity when the daunorubicin-resistant cell line, P388/PR8/22, was compared with the parent cell line, P388. However this increase was not significant.

We could not demonstrate any significant degree of cross-resistance towards daunorubicin in the bryostatin 1-resistant cell lines. Equally, bryostatin 1 demonstrates similar growth-inhibitory effects on both P388 and the daunorubicin-resistant subline. We conclude that bryostatin 1 is not one of the 'mdr' class of agents. This is in agreement with Hait and De Rosa (1991), who have shown that a subclone of HL-60 human promyelocytic leukaemia with acquired resistance to phorbol esters showed no change in sensitivity to doxorubicin.

In conclusion, this work has shown that the relationship between levels of PKC activity and sensitivity towards bryostatin 1 or phorbol esters is a complex one. However, continuous exposure of cells to bryostatin 1 results in the rapid development of a stable cell line with decreased total PKC activity. The importance of this residual PKC activity to cell proliferation has yet to be determined.

This work was supported by the Cancer Research Campaign. The cell culture skills of Tim Ward and Sally Haran are acknowledged.

### References

AZZI, A., BOSCOBOINIK, D. & HENSEY, C. (1992). The protein kinase C family. *Eur. J. Biochem.*, **208**, 547–557.

BERKOW, R.L. & KRAFT, A.S. (1985). Bryostatin, a non-phorbol macrocyclic lactone, activates intact human polymorphonuclear leukocytes and binds to the phorbol ester receptor. *Biochem. Biophys. Res. Commun.*, **131**, 1109–1116.

BLUMBERG, P.M. & PETTIT, G.R. (1992). The bryostatins, a family of protein kinase C activators with therapeutic potential. *New Leads and Targets in Drug Research. Alfred Benzon Symposium*, **33**, 273–285.

CASTAGNA, M., TAKAI, Y., KAIBUCHI, K., SANO, K., KIDDAWA, U. & NISHIZUKA, Y. (1982). Direct activation of calcium-activated, phospholipid-dependent protein kinase by tumour promoting phorbol esters. *J. Biol. Chem.*, **257**, 7847–7851.

DALE, I.L. & GESCHER, A. (1989). Effects of activators of protein kinase C, including bryostatins 1 and 2 on the growth of A549 human lung carcinoma cells. *Int. J. Cancer*, **43**, 158–163.

FIELDS, A.P., PETTIT, G.R. & MAY, W.S. (1988). Phosphorylation of laminin B at the nuclear membrane by activated protein kinase C. *J. Biol. Chem.*, **263**, 8253–8260.

GESCHER, A. (1992). Towards selective pharmacological modulation of protein kinase C – opportunities for the development of novel antineoplastic agents. *Br. J. Cancer*, **66**, 10–19.

HAIT, W.N. & DE ROSA, W.T. (1991). The role of the phorbol ester receptor protein kinase C in the sensitivity of leukemic cells to anthracyclines. *Cancer Commun.*, **3**, 77–81.

HANNUM, Y.A. & BELL, R.M. (1987). Mixed micellar assay for phorbol ester binding. *Methods Enzymol.*, **141**, 287–293.

HENNINGS, H., BLUMBERG, P.M., PETTIT, G.R., HERALD, C.L., SHORES, R. & YUSPA, S.H. (1987). Bryostatin 1, an activator of protein kinase C, inhibits tumor promotion by phorbol esters in SENCAR mouse skin. *Carcinogenesis*, **8**, 1343–1346.

HORNUNG, R.L., PEARSON, J.W., BECKWITH, M. & LONGO, D.L. (1992). Preclinical evaluation of bryostatin as an anticancer agent against several murine tumor cell lines: *in vitro* versus *in vivo* activity. *Cancer Res.*, **52**, 101-107.

JETTEN, A.M., GEORGE, M.A., PETTIT, G.R. & REARICK, J.I. (1989). Effects of Bryosatin and retinoic acid on phorbol ester and diacylglycerol induced squamous differentiation in human tracheobronchial epithelial cells. *Cancer Res.*, **49**, 3990-3995.

KISS, Z., RAPP, V.R., PETTIT, G.R. & ANDERSON, W.B. (1991). Phorbol ester and bryostatin differentially regulate the hydrolysis of phosphatidylethanolamine in Ha-ras and -raf-oncogene transformed NIH3T3 cells. *Biochemistry*, **276**, 505-509.

KONIG, B., DI NITTO, P.A. & BLUMBERG, P.M. (1985). Phospholipid and $Ca^{++}$ dependency of phorbol ester receptors. *J. Cell. Biochem.*, **27**, 255-265.

KRAFT, A.S., SMITH, J.B. & BERKOW, R.L. (1986). Bryostatin, an activator of the calcium phospholipid-dependent protein kinase, blocks phorbol ester-induced differentiation of human promyelocytic leukaemia cells HL-60. *Proc. Natl Acad. Sci. USA*, **83**, 1334-1348.

McGOWN, A.T., WARD, T.H. & FOX, B.W. (1983). Comparative studies of the uptake of daunorubicin in sensitive and resistant P388 cell lines by flow cytometry and biochemical extraction procedures. *Cancer Chemother. Pharmacol.*, **11**, 113-116.

NAKADATE, T. & BLUMBERG, P.M. (1987). Modulation by palmitoylcarnitine of protein kinase C activation. *Cancer Res.*, **47**, 6537-6542.

NELSESTUEN, G.L. & BAZZI, M.D. (1991). Activation and regulation of protein kinase C enzymes. *J. Bioenerg. Biomembr.*, **23**, 43-61.

NISHIZUKA, Y. (1988). The molecular heterogeneity of protein kinase C and its implications for cellular regulation. *Nature*, **334**, 661-665.

PALAYOOR, S.T., STEIN, J.M. & HAIT, W.N. (1987). Inhibition of protein kinase C by antineoplastic agents: implications for drug resistance. *Biochem. Biophys. Res. Commun.*, **148**, 718-725.

PARKER, P.J., KOUR, G., MARAIS, R.M., MITCHELL, F., PEARS, C.J., SCHAAP, D., STABEL, S. & WEBSTER, C. (1989). Protein kinase C - a family affair. *Mol. Cell. Endocrinol.*, **65**, 1-11.

PETTIT, G.R., HERALD, C.L., DOUBEK, D.L. & HERALD, D.L. (1982). Isolation and structure of bryostatin 1. *J. Am. Chem. Soc.*, **104**, 6846-6848.

SAKO, T., YUSKA, S.H., HERALD, C.L., PETTIT, G.R. & BLUMBERG, P.M. (1987). Partial parallelism and partial blockade by bryostatin 1 of effects of phorbol ester tumor promoters on primary mouse epidermal cells. *Cancer Res.*, **47**, 5445-5450.

SMITH, J.B., SMITH, L. & PETTIT, G.R. (1985). Bryostatins: potent, new mitogens that mimic phorbol ester tumor promoters. *Biochem. Biophys. Res. Commun.*, **132**, 939-945.

YASUDA, I., KISHIMATO, A., TANAKA, S., TOMINAGA, M., SAKURAI, A., NISHIZUKA, Y. (1990). A synthetic peptide for selective assay of protein kinase C. *Biochem. Biophys. Res. Commun.*, **166**, 1220-1225.